# Biological Characteristics and Rearing Techniques for Vespid Wasps with Emphasis on *Vespa mandarinia*

**DOI:** 10.3390/insects16121231

**Published:** 2025-12-06

**Authors:** Lijuan Lv, Juan Du, Guoliang Wei, Yu Tian, Shangwei Li

**Affiliations:** 1 Guizhou Key Laboratory of Agricultural Biosecurity, Institute of Entomology, Guizhou University, Guiyang 550025, China; lvlj2025@163.com (L.L.); juandudj@163.com (J.D.); 2 Rongjiang County Guoliang Wasp Breeding Farmers’ Professional Cooperative, Bakai Town, Rongjiang County, Qiandongnan Miao and Dong Autonomous Prefecture 556000, China; wgl57688881@163.com; 3 Guizhou Fanjingshan National Nature Reserve Administration, Jiangkou County, Tongren 554400, China

**Keywords:** vespid wasps, *Vespa mandarinia*, biological characteristics, artificial indoor rearing

## Abstract

Vespid wasps (Hymenoptera: Vespidae) represent ecologically and economically significant insect resources, possessing extremely high nutritional and medicinal value. In response to escalating market demand and declining wild populations, artificial indoor rearing of vespid wasps has emerged as a sustainable strategy. This approach not only helps conserve and utilize this valuable resource, supporting traditional medicine and local cuisine, but also contributes to invasive species control in affected regions. The large-scale rearing of wasps must integrate their biological characteristics with advanced rearing technologies to achieve rational rearing practices, while continuously optimizing management models. This article systematically reviews the biological characteristics of wasps, traditional rearing methods, and the current status of wasp rearing. It focuses on the methods and key technologies for the outdoor rearing of *Vespa mandarinia* and year-round indoor rearing. Additionally, it discusses the advantages of artificial indoor rearing, identifies critical technical challenges, and provides a summary and outlook on the future development trends, aiming to provide theoretical support for the large-scale and industrialized development of indoor wasp rearing.

## Simple Summary

This paper systematically reviews the biological characteristics of wasps, traditional rearing methods, and the current status of wasp rearing, with a focus on the methods and key technologies for the outdoor rearing of *Vespa mandarinia* and year-round indoor rearing. Additionally, the paper summarizes and prospects the future development trends, aiming to provide theoretical references for the large-scale and industrial development of artificial indoor wasp rearing.

## 1. Introduction

Vespid wasps, commonly known as hornets or yellow jackets, are a general term for insects of the family Vespidae, order Hymenoptera, class Insecta, within the phylum Arthropoda. This insect serves as both food and medicine. The wasps is a treasure trove of resources; its pupae, larvae, adult, venom, and nest are all commonly used in traditional Chinese medicine. Wasps are used in traditional medicine to treat heart and abdominal distension and pain, retching, reducing bodily heaviness and replenish qi (vital energy), dispelling wind and detoxification, as well as freckles and facial sores [1]. Wasp wine, made by steeping wasps in wine, shows particularly outstanding effectiveness in treating rheumatism [2]. The 2025 edition of the Chinese Pharmacopoeia records that wasp nest has the efficacy of detoxification, insecticidal, dispelling wind and relieving pain [3]. Wasp venom exhibits antitumor, analgesic, and anti-inflammatory effects [4,5]. China has a long history of consuming wasps, with wasp pupae and larvae being the most commonly eaten insects in regions such as Yunnan and Southeast Guizhou. Internationally, countries such as Japan, Mexico, and Thailand also have traditions of consuming wasps [6,7,8]. Wasps are rich in protein, amino acids, mineral elements, unsaturated fatty acids, and trace elements. As a high-protein, low-fat food source, they help prevent iron-deficiency anemia and delay aging effects, making them an excellent nutritional and health food [9]. Wasps can also be utilized as effective predatory insects, preying on a variety of forestry and grassland pests, such as the *Cnaphalocrocis medinalis* (Guenée) (Lepidoptera: Crambidae), *Dendrolimus tabulaeformis* Tsai & Liu (Lepidoptera: Lasiocampidae), and *Helicoverpa armigera* (Hübner) (Lepidoptera: Noctuidae) [10]. For example, it has been reported that in Shimen County, Hunan Province, China, the use of wasps for the biological control of *H. armigera* saved one township 127,600 CNY in expenses [11]. Wasps feed on nectar and contribute to wild plant pollination, playing a particularly important role in the pollination of *Camellia sinensis* Kuntze (Theaceae) and *Codonopsis pilosula* (Franch.) Nannf. (Campanulaceae) [12].

Wasps are highly aggressive social insects whose ferocity far exceeds that of honeybees. As voracious predators of honeybees, they pose severe threats to apiculture and pollination ecosystems [13,14]. During the peak activity period of wasps in autumn, they frequently swarm and attack humans, causing localized skin erythema, edema, and pain. In severe cases, allergic reactions and multiple organ dysfunction may occur, potentially leading to death [15,16]. Wasp rearing demonstrates dual value in ecological conservation and economic development. On one hand, invasive wasps pose a substantial threat to apiculture. As a result, effective management strategies to mitigate the impact of invasive wasps on apiculture have become a global concern. On the other hand, wasp rearing has the potential to be integrated into sustainable insect rearing systems. Perrard et al. conducted research in France on the rearing of the invasive wasp *Vespa velutina* Lepeletier to control its spread [17]. In the context of sustainable agriculture and the utilization of insect resources, wasps can serve as potential biological control agents and sources of protein, making their farming of considerable research and application value. Exploring the integration of wasp rearing into sustainable insect rearing systems could provide new approaches to addressing global food security and environmental protection issues. Traditional wasp cultivation frequently fails due to inadequate understanding of wasp biological characteristics, particularly regarding wasp rearing management, the principles of feed management, and integrated pest management [18]. Therefore, this paper provides a comprehensive overview of wasp biological characteristics, traditional rearing methods, and the current status of wasp rearing, with a focus on the methods and key technologies for the outdoor rearing of Asian giant hornet *Vespa mandarinia* and year-round indoor rearing.

## 2. Biological Characteristics of Vespid Wasps

### 2.1. Classification and Distribution

The family Vespidae encompasses over 5000 described species worldwide, distributed in China, India, Japan and other regions. Currently, over 200 species have been documented in China, primarily distributed in Yunnan, Anhui, Beijing, Guizhou and Sichuan [16,19]. This wasp species primarily inhabits mountainous areas with an altitude of 850–1900 m, typically establishing nests in decaying underground root systems or abandoned rodent burrows [20]. The family Vespidae comprises six subfamilies: Vespinae, Polistinae, Eumeninae, Euparagiinae, Masarinae, Stenogastrinae [21]. The Euparagiinae subfamily represents the most primitive lineage within Vespidae, containing only a single genus with 10 species, distributed in the arid regions of the southwestern United States, Mexico, and parts of China. Eumeninae is the largest subfamily, containing 203 genera and approximately 3000 species distributed worldwide [22]. Stenogastrinae is restricted to the Oriental region, comprising 6 genera and approximately 50 species. Polistinae, a common subfamily, exhibits a global distribution with 28 genera and 800 species. Vespinae currently comprises 4 genera with 60 species, its primary distribution centered in the Northern Hemisphere and tropical Asia. Masarinae comprises 12 genera and 299 described species with a global distribution, predominantly found in the Afrotropical, Palearctic, Australasian, Neotropical, and Nearctic regions [23,24,25]. Wasp rearing is primarily focused on social wasps, especially those belonging to the subfamily Polistinae and Vespinae. This is because they can form large colonies, producing sufficient biomass (larvae, pupae) and thus holding high economic value. The requirements for the environment differ significantly between the subfamilies Polistinae and Vespinae. Wasps of the subfamily Polistinae typically build nests in open areas, such as tree branches and under eaves, and have a strong adaptability to the environment. In contrast, wasps of the subfamily Vespinae prefer to build nests at the edge of forests or in shrubs, and have higher requirements for the concealment and humidity of the environment. Therefore, it is recommended that the breeding populations should preferably use local indigenous sources as much as possible. Currently, the most productive species in China’s wasp rearing industry are *V. mandarinia*, *Vespula vulgaris* (Linnaeus), and *Vespa basalis* Smith, followed by *Vespa ducalis* Smith and *V. velutina* [12].

### 2.2. Morphological Characteristics

**Figure 1:**
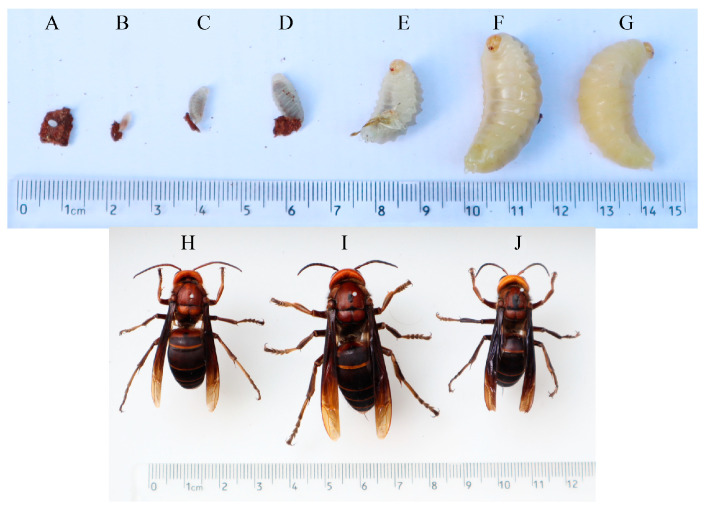
The life history of *V. mandarinia*. (**A**) Egg; (**B**–**F**) First- to fifth-instar larvae; (**G**) Pupa; (**H**) Drone; (**I**) Queen; (**J**) Worker.

The life history of wasps encompasses four distinct stages: egg, larva, pupa, and adult, which is characteristic of holometabolous insects, as shown in Figure 1. The eggs are elongate oval, white, and attached individually to the nest wall. Larvae are legless, spindle-shaped, and white, ranging from 7–35 mm in length. They can turn their heads slightly and spin silk by themselves. After the nest cell is sealed, they pupate. The pupal stage lasts for about 6 days. The pupa undergoes metamorphosis into an adult wasp within the nest cell, and the adult wasp chews through the nest cell to emerge. The body length of adult wasps of most species is concentrated in the range of 20–45 mm. It is commonly black and yellow striped or entirely black. The head is slightly flattened at the front and back. It has compound eyes and antennae, as well as chewing mouthparts. Both the mesothorax and metathorax have a pair of membranous wings. Legs are robust and multifunctional, used in prey capture, nest construction, and feeding.

Sexual dimorphism is marked: males possess 13 antennal segments, often with a hooked terminal segment, and 7 visible abdominal segments, ending in a slender gonostylus. Females have 12 antennal segments and 6 abdominal segments, with a modified ovipositor connected to venom glands, enabling stinging behavior [26]. The completion of a generation of wasps typically requires about 40–44 days.

### 2.3. Live Habits

#### 2.3.1. Social Organization and Its Rearing Implications

Wasps are typical eusocial insects that live in colonies. A wasp colony is composed of a queen, workers (sterile females), and males. The colony is centrally governed by the queen, whose egg-laying capacity directly determines population growth and productivity. In rearing systems, safeguarding the queen and maintaining its physiological health are of paramount importance. Workers exhibit age-based polyethism. Young workers are only responsible for lighter tasks such as cleaning, which are mostly performed near the nest, while adult workers take on heavier tasks outside the nest, such as foraging, feeding larvae, defending against enemies, and protecting the nest. Male wasps only appear during the mating season, primarily to mate with the queen [27]. In controlled rearing, managing this reproductive timing helps optimize resource allocation and prevents unnecessary competition. The social life cycle of wasps is divided into six periods: pre-nesting period, solitary period, cooperative period, polyethic period, mating period, hibernating period [7]. In artificial indoor rearing systems, implementing such a life cycle-based precision management strategy with targeted human intervention can effectively stabilize colony development trajectories and significantly enhance both queen oviposition efficiency and overall rearing productivity.

#### 2.3.2. Nesting Behavior and Artificial Nest Design

The nest is the core of the wasp colony’s survival. Nesting behavior is critical to guiding the design of artificial nesting structures in wasp rearing. Wasps prefer concealed locations such as grasses, tree tops, tree cavities, caves, and rock crevices, highlighting the importance of providing dark, enclosed, and low-disturbance nesting sites in artificial rearing. The nests are made by workers chewing plant fibers and mixing them with saliva. In the initial stage, the overwintered queen selects a suitable nesting site and independently constructs the pedicel. Subsequently, new cells are progressively added to the outer sides between the existing cells. This process indicates that artificial nest setups should include suitable attachment points and adequate fibrous materials to stimulate natural building behavior. After feeding, the larvae secrete protein liquid substances that attract adult individuals, which stimulate workers to go out more frequently to find nesting materials [28]. Therefore, in intensive rearing systems, providing protein supplements may reduce the workers’ energy expenditure required for foraging for nest-building materials, thereby increasing their efficiency. Nests of *V. mandarinia* under artificial rearing can attain weights up to 7.5 times greater than those of wild nests [7]. Furthermore, seasonal nest migration behavior suggests that successful year-round indoor rearing requires simulating environmental cues, such as temperature and light cycles, to prevent unwanted colony relocation or nesting failure in wasps.

#### 2.3.3. Predatory Behavior and Feeding Management

As predatory insects, wasps primarily hunt Lepidoptera and Diptera larvae, showing a strong preference for live prey. This behavior supports their use as biological control agents in integrated pest management systems [24]. However, in artificial rearing, it is essential to develop efficient feeder-insect production or protein-substitute formulas to ensure a stable and cost-effective diet. Wasps are also influenced by weather and temperature, with optimal foraging activity occurring between 23 °C and 30 °C. This suggests that indoor rearing environments should maintain temperatures within this range to promote feeding and hunting behaviors. In periods of food scarcity, wasps may resort to cannibalism [29], underscoring the importance of a consistent and adequate nutritional supply in artificial rearing. Additionally, adult wasps consume carbohydrate sources such as nectar and fruit, indicating that artificial diets should include sugar-based solutions and fruit purees to support energy needs. The demonstrated learning and memory capacities of wasps regarding visual and olfactory cues can be leveraged to train them to accept artificial diets or utilize optimized feeding stations [30]. For instance, studies have shown that wasps can learn and remember visual and olfactory cues associated with food sources, which can influence their foraging behavior [31].

#### 2.3.4. Reproductive Cycle and Colony Population Control

New queen wasps usually mate only once, with the mating process typically lasting for several minutes. After mating, the sperms are stored in the queen’s spermatheca, where they can remain viable for up to 300 days and are utilized progressively for fertilization during oviposition. After mating, they overwinter in safe places such as tree holes and wall crevices. Therefore, this behavior must be replicated in rearing systems using climate-controlled overwintering units that provide dark, enclosed, and low-disturbance environments to ensure queen survival and successful colony initiation in spring. The duration of hibernating is influenced by the altitude of the habitat. In tropical and subtropical regions, founding queens do not overwinter. They can establish nests individually throughout the year [32]. Consequently, controlled regulation of temperature and humidity in indoor rearing systems can facilitate year-round colony maintenance. The rapid colony expansion from July to August, followed by the production of new queens and males in autumn, defines the most productive phase in rearing. To maximize yields, nutritional and spatial resources should be scaled up during this period. Wasps in warm and humid regions exhibit faster colony development, larger nest sizes, and longer reproductive seasons, whereas those in cold and arid areas show slower growth, smaller colonies, and shorter rearing periods [33]. Thus, the introduction of mated queens into pre-established nesting environments in early spring can synchronize colony development and support large-scale, standardized production.

#### 2.3.5. Characteristics of *V. mandarinia*

*V*. *mandarinia*, also known as the tiger-headed hornet, banded hornet, or bull-horn hornet, is the world’s largest vespine species. Queens can reach up to 50 mm in body length, while workers typically measure 40 mm [34]. The head is orange-yellow, and the abdomen exhibits distinct alternating black and yellow bands. This species is native to East and Southeast Asia, where it inhabits low-altitude forests, farmlands, and village peripheries. Nests are typically constructed in subterranean cavities or tree hollows. *V. mandarinia* is a polyphagous predator with a strong carnivorous tendency. It preys on a diverse array of insects and has a devastating impact on regional beekeeping. *V. mandarinia* is considered a high-risk species due to its formidable stinger and defensive aggression. A single sting can deliver a large bolus of a complex venom, posing a potentially lethal threat to humans and livestock [35,36]. The pupae of *V. mandarinia* are a high-protein food source. Therefore, establishing standardized indoor rearing system is crucial for their sustainable utilization and for mitigating ecological impacts from wild collection [37].

## 3. Rearing Technology of *V. mandarinia*

Wasp culture targets the sustainable production of pupae, venom, and wasps wine; thus, a scientifically grounded rearing protocol must be established. The current rearing method for *V. mandarinia* typically commences with the establishment of a small wasp colony, comprising approximately 15 wasps. Subsequently, these wasps are released into the wild. This practice is cost-effective and can result in high yields of wasp larvae, providing wasp keeping with significant economic benefits. However, outdoor rearing practices may disrupt ecological equilibrium and pose potential risks to human and livestock safety. Studies have demonstrated that during the outdoor rearing process of wasps, genetic exchange has occurred with local wild natural populations or introduced populations, resulting in differences in genetic diversity among various populations [38]. Therefore, it is recommended that breeding populations utilize local indigenous sources as much as possible to minimize the impact of foreign sources on the local ecosystem. Meanwhile, it is crucial to strictly prevent the escape of introduced reared individuals, which could interbreed with wild populations and potentially lead to an increase in the genetic diversity of local wild populations. Therefore, the future direction of rearing *V. mandarinia* is to shift from wild release to full-indoor artificial rearing in greenhouses, thereby achieving industrial-scale production.

### 3.1. Pre-Rearing Preparations

#### 3.1.1. Site Selection

The ideal site for *V. mandarinia* rearing should be situated in a location that is sheltered from the wind and faces the sun, with rich plant diversity, abundant sunlight and rainfall, and a plentiful supply of insect resources to ensure the availability of the food sources required by wasps, such as insects, tree sap, and nectar. The site should also be located away from residential areas to avoid noise, pesticide contamination, and animals that pose a threat to wasps [24,26]. The wild rearing farms for *V*. *mandarinia* are equipped with trees that provide materials for building nests, such as fir trees, pine trees, or decaying wood. Prominent warning signs for wasps should be hung around the wild rearing farms or rearing boxes to prevent unauthorized personnel from entering.

#### 3.1.2. Main Rearing Facilities and Equipment

Establish a rearing base at a location that is a moderate distance from the wild rearing farm and has relatively convenient transportation. The base should include a mating room, a overwintering room, and a small wasp colony rearing room. The top and sides of the mating room are covered with insect-proof netting. The interior should be equipped with flowers and sugar water to provide adequate nutrition for adults. Maintain temperatures at 20–30 °C with 50–70% relative humidity [7,27]. These conditions provide sufficient mating time and opportunities for the males and gynes, ensuring adequate fertilization of the queen and reducing the occurrence of inferior queens. The traditional overwintering room is made of carved-out wooden log halves. It is situated in a shaded area, with the substrate kept moist, a temperature of 0–15 °C, and a humidity of 60–80% [7]. This method results in a high adult survival rate during overwintering (up to 90%). However, a substantial mortality rate (exceeding 60%) is commonly observed after the overwintering period concludes [18]. Currently, low-temperature overwintering is widely adopted. The queens are placed in wooden boxes, with one side of the box covered with iron wire netting. Typically, 300 queens are housed in a wooden box measuring 30 cm × 30 cm × 50 cm. These boxes are then stored in a refrigerator set at a temperature of 4–5 °C. The survival rate of the queens after overwintering can reach 97% [13]. In the small wasp colony rearing room, box racks are placed, with nest boxes situated on these racks. The temperature is maintained at 20–30 °C, and the humidity is kept at 60–80%. Field rearing boxes (1 m × 1 m × 1 m), air conditioners, humidifiers, hygrometers, electroshock venom extractors, beekeeping suits, and wasp first aid kits are also necessary.

### 3.2. Rearing Technology and Management

#### 3.2.1. The Acquisition of Prospective Queen Wasps

In early November each year, prospective queens and males from the nest boxes are collected using wasp collection cages and then transported to the mating room at the rearing base. To avoid long-term closed rearing degeneration of wasp colonies, it is necessary to collect wild queens annually or appropriately introduce high-quality wasp colonies from other regions for hybridization and breeding improvement.

#### 3.2.2. Mating Period Management

To enhance the quality of the queens and avoid inbreeding, potential queens and males from different locations are centrally placed into a mating room for unified rearing, thereby increasing the number of individuals. Both natural mating and artificial mating assistance methods are employed. Artificial assisted mating can address issues of insufficient mating. Intervening in the mating process to prevent the gynes from biting the males ensures that the males can mate multiple times, allowing the gynes to receive sperm from more males. This also increases the genetic diversity of the offspring. In the natural environment, approximately 8% of nests belonging to the Vespinae subfamily are usurped. Thus, the practice of isolating a single queen for rearing can effectively prevent usurpation by conspecific and heterospecific queens [39]. The mating room should provide ample nectar, sugar water, 0.2% complex vitamins, and insect feed that is high in protein. At the same time, pay attention to environmental sanitation, routine disinfection, and preventing the introduction of external pathogens.

#### 3.2.3. Management During the Expansion Period of Small Wasp Colonies

**Figure 2:**
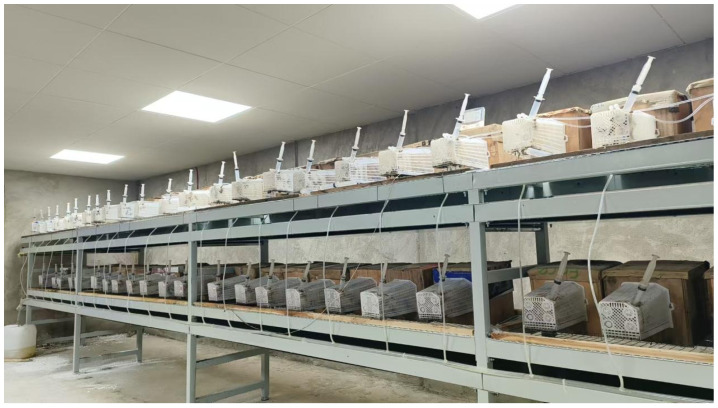
Rearing room for small colonies of *V. mandarinia*.

Generally, fertilized queens are placed into refrigerators in December for overwintering. In early March of the following year, the overwintered queens are transferred to small wasp colony rearing room to be awakened. The small wasp colony rearing room is shown in Figure 2. After the queen awakens, it requires a period of recovery during which it needs to be provided with a variety of foods, including honey water, bees, locusts, mealworms, various lean meats, and mineral elements. When the queen becomes actively engaged in flight and begins to exhibit combative behavior with other wasps, it should be immediately transferred to the nest box of a small wasp colony rearing device, which measures 15 cm × 15 cm × 15 cm. A transverse wooden bar is installed at the top of the nest box, with a nest base mounted at its center, and the nest cell opening facing downward. The nest base is constructed using 3D-printed fir bark, wheat straw, or corn stalks. The queen begins constructing the nest on the nest base using fir bark placed inside the nest box. After the first nest cell is built, the queen lays a single egg inside it. Before the egg hatches, the queen continues to construct nest cells in a circular pattern, laying eggs as it builds. After the eggs hatch into larvae, the queen, in addition to nest building and egg-laying, also takes on the responsibility of feeding the larvae. The first batch of eggs, after approximately one month, will eclose into the first cohort of workers, which then assume the responsibilities of nest building and larval feeding. At this point, the queen’s sole responsibility is oviposition. The side wall of the nest box features a round hole that connects to a feeding area. The feeding area is equipped with syringes that provide honey and water. Within the feeding area, locusts are offered as food for the wasps, or alternatively, artificial feed is provided. When the number of workers in the nest box reaches 15, the nest box can be relocated to a larger field hive for further development.

#### 3.2.4. Field Rearing Management

**Figure 3:**
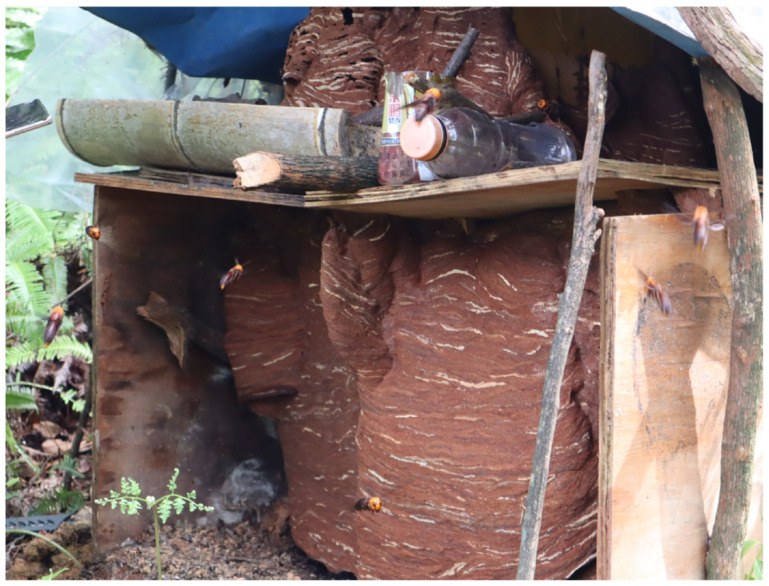
The nest of field-reared *V. mandarinia*.

The field hive is placed in the forest. Field nest hives are shown in Figure 3. The lower part of the hive has small holes, and the bottom is open, placed directly on the ground. The rearing hive is covered with asbestos tiles on top to protect against rain and direct sunlight. Drainage ditches are dug on both sides of the hive to prevent water accumulation at the bottom. The nest from the nest box is placed within the rearing hive and secured at the top of the hive structure, ensuring the integrity of the nest’s outer shell. The distance between rearing hives should ideally be more than 1 km. Warning signs for wasps should be hung near the rearing hives. When a small wasp colony is first moved to the field, placing food such as honey water, live insects, meat, or artificial feed near the hive can facilitate colony survival. During the rearing period, it is advisable to continue placing food near the hives to meet the nutritional requirements of the wasps, which can enhance the yield of wasp pupae.

#### 3.2.5. Year-Round Indoor Rearing

**Figure 4:**
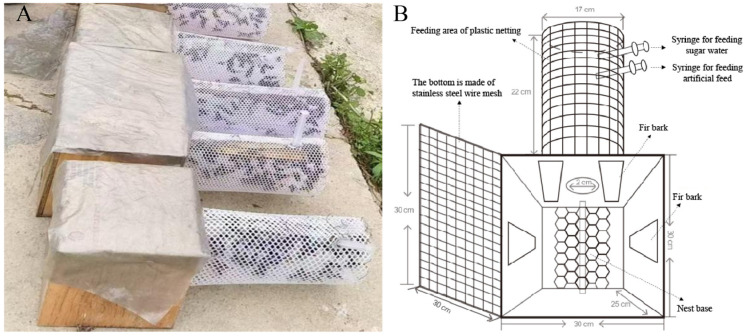
The box for indoor-reared *V. mandarinia*. (**A**) The actual rearing box; (**B**) Structural diagram of the rearing box. The rearing box features a stainless-steel wire mesh at the bottom of the nest box, a 3D-printed nest base at the top, and the provision of fir bark. The nest box is connected at one side to a plastic mesh feeding area, where two syringes are inserted to supply sugar water and artificial feed, respectively.

**Figure 5:**
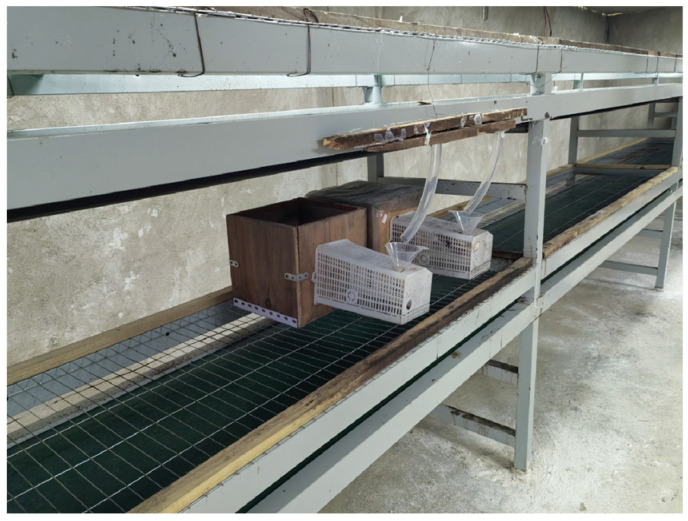
Large-scale indoor rearing system for *V. mandarinia*.

An indoor rearing room for the wasps is established at the rearing base, equipped with rearing box racks. The indoor temperature is maintained at 26 °C ± 2 °C, and the humidity is kept at 75% ± 2%. The rearing box is similar to a small wasp colony rearing device, but it is larger in size (30 cm × 30 cm × 25 cm). The nest box is shown in Figure 4. A conveyor belt is installed between the bottom of the rearing box and the rearing box rack, facilitating the removal of feces produced by the wasps. The feeding area connected to the rearing box is equipped with funnels, which are connected to tubes. This setup facilitates the delivery of artificial feed and honey water through vacuum pump gas transport within the tubes, achieving automated feeding and feces removal. In a rearing box, wasps can construct up to four layers of combs. After harvesting the pupae (approximately 3 kg), the rearing process can continue. The entire rearing process is conducted indoors, eliminating the need for field rearing. A year-round indoor rearing system is shown in Figure 5. Two key technologies for indoor rearing are the artificial feed for wasps and pest control agents. The differences between field rearing and artificial indoor rearing modes are shown in Table 1.

### 3.3. Feed Production Technology

The feeding principle for wasp rearing is to primarily use natural insects during the larval stage, supplemented by artificial feed to ensure a balanced diet. During the adult stage, the frequency of sugar water feeding should be increased to reduce the energy expenditure of the wasps.

#### 3.3.1. Artificial Feed

Artificial feed for wasp rearing has several advantages, including being unaffected by weather conditions, avoiding interference from parasitism and predation, and having stable nutritional components that can be controlled in terms of formulation. Therefore, it has a good application prospect in wasp rearing. Artificial feed for wasps is primarily composed of nutrients, shaping agents, feeding stimulants, and preservatives. Nutrients include carbohydrates, fatty acids, proteins, amino acids, mineral elements, and complex vitamins, which are the various nutritional substances required for the growth and development of wasps. The shaping agents help maintain the shape of the feed and regulate its physical properties. The feeding stimulants can stimulate and promote wasp feeding. Preservatives inhibit microbial growth to prevent diet spoilage. Miaoqi Biotechnology Company provides an artificial feed for wasps, which is composed of the following ingredients by weight: 5–10 parts honey, 10–30 parts fresh pears, 10–20 parts peaches, 5–10 parts white sugar, 5–10 parts soybean meal, 0.1–2 parts complex vitamins, 5–10 parts mealworm larvae, and 5–20 parts water. The aforementioned ingredients are blended and ground into a pulp [40].

#### 3.3.2. Natural Feed

Rearing of yellow mealworms: The yellow mealworms primarily feed on wheat bran and vegetables. They thrive at a temperature of 25–30 °C and a relative humidity of 60–70%. When the larvae grow to 2–3 cm in length, they can be used as feed [41]. The adult survival period is approximately 50 days, and they serve as a major protein source for wasp larvae.

Rearing of locusts: Locusts primarily feed on wheat seedlings, corn seedlings, and weeds. The suitable rearing conditions are a temperature of 25–30 °C, a relative humidity of approximately 85%, and 14–16 h of daily light exposure. It is essential to maintain a clean rearing environment. After about 35 days, the locusts reach maturity, with their body weight and nutritional content reaching higher levels, making them suitable for use as wasp feed.

Rearing of honeybees: Italian honeybees or Chinese honeybees can be reared, and eliminated workers and males should be collected. Care should be taken to avoid interspecific conflicts between wasps and honeybees during rearing.

## 4. Natural Enemies and Pest Control of Wasps

### 4.1. Main Natural Enemies of Wasps

Avian predators such as magpies, crows, and sparrows prey on both adults and larvae, necessitating the installation of anti-bird nets in rearing areas. Parasitic organisms, primarily parasitoid wasps and pyralid moths, require regular nest inspections and removal to maintain colony health. Frogs and toads are known to prey on wasps. To prevent such predation on wasps, it is advisable to elevate the hives. Other insects such as spiders and ants are known to prey on the larvae and eggs of wasps. To prevent this predation, it is recommended to apply a layer of petroleum jelly to the bottom of the hives. Alternatively, the hives can be elevated using iron frames, with bottles placed around the legs of the frames. A small amount of waste engine oil can be placed inside these bottles to deter ants from entering the hives.

### 4.2. Main Pests and Diseases of Wasps

The primary source of sacbrood disease is the diseased larvae, and the virus is mainly transmitted to healthy larvae through the feeding activities of workers. Transmission occurs via the oral route, with the virus entering the larval body through ingestion of contaminated food. By intervening in the reproductive cycle of wasp colonies, timely queen replacement and brood room cleaning can be carried out to reduce the number of virus hosts and break the cycle of transmission, thereby reducing the main source of infection. In terms of treatment, using 50 g of dried *Scutellaria barbata* D.Don (Lamiaceae), decoct it in water to prepare a soup. This herbal preparation can be used to treat 20 to 30 frames of wasps [35].

Larval foulbrood diseases and bacterial diseases often occur in larvae and pupae. During the hot and rainy humid season, when there are many larvae in the comb and the brood-rearing task is heavy, the disease is more likely to occur. After infection, the insect body turns black, emits a foul smell, and spreads very quickly. In severe cases, the brood comb exhibits a “pepper-box” pattern, leading to rapid colony collapse due to the extensive death of larvae and pupae. To control the disease, it is necessary to promptly remove the diseased combs and disinfect them strictly. Antibiotics can be applied to other combs for disinfection and sterilization. For early prevention, combs should be dispersed, feed disinfection should be enhanced, and antibiotics can be sprayed on the combs. For the treatment regimen, 10 g of *Scutellaria baicalensis* Georgi (Lamiales: Lamiaceae) and 15 g of *Coptis chinensis* Franch. (Ranunculales: Ranunculaceae) are combined. These herbs are decocted in 250 mL of water until the volume is reduced to 150 mL. The resultant liquid is then used for spraying the combs after the wasps have been removed. This process is carried out every other day for a total of three times [35].

Parasitic diseases, such as those caused by the *Hypsopygia postilava* (Hampson) (Lepidoptera: Pyralidae), can be particularly damaging to wasp colonies. Adult females of *H. postilava* lay eggs on the comb at night. After 4–5 days, the eggs hatch into larvae, which then feed on the larvae of wasps, causing the disintegration of the comb. To control this disease, it is necessary to construct the wasp nests at a height of more than 2 m above the ground to reduce the damage caused by the *H. postilava*. Within 2 to 3 days after the *H. postilava* lays eggs, the comb entrance should be closed every night to prevent the adult *H. postilava* from entering and laying eggs.

### 4.3. Integrated Control Methods

In the management of diseases, particularly for wasps, it is essential to identify and target the critical stages of disease development when designing control strategies. Only through the implementation of integrated control methods can diseases be effectively managed and essentially contained. The implementation of pest management strategies for vespid wasps can be categorized into four major types: pest and disease quarantine, breeding of disease-resistant wasp strains, management techniques for prevention, and chemical control [35].

#### 4.3.1. Pest, Disease Quarantine, and Chemical Control

The management of wasp pests and diseases should strictly adhere to the principle of “prevention first”. Prior to rearing, the site should be thoroughly disinfected by spraying with a 5% solution of lime water or by spreading wood ash. Overwintering rooms can be disinfected by spraying with a 5% bleach solution. It is important to locate the rearing site away from sewage pits and near a clean water source, with attention to proper ventilation and maintain good environmental sanitation. Feed and wasp equipment can be disinfected using methods such as sulfur fumigation, acetic acid fumigation, and ethylene oxide fumigation. When disinfecting wasp equipment, it is essential to remove the wasps, ensuring that there is no pollen or honey present to prevent wasp loss and to enhance the effectiveness of the disinfection. Strict quarantine procedures should be implemented when introducing new wasp colonies. In vespid wasp rearing, the use of chemical agents should be carried out with caution, only when necessary and based on specific circumstances, and strictly in accordance with relevant regulations and guidelines. The method of prevention and control involves adding appropriate drugs to the sugar syrup that is fed to the wasps to prevent common diseases. The “capture and medicate method” can also be employed, where wasps carrying the medicine bring the agent back to the nest, thereby effectively controlling pathogens within the nest. In wasp rearing, chemical control should be combined with measures such as hive replacement, comb replacement, and disinfection. Before chemical control, it is imperative to eliminate the sources of infection, such as by collecting and killing diseased wasps, in order to achieve satisfactory therapeutic outcomes.

#### 4.3.2. Breeding of Disease-Resistant Wasp Strains

In the rearing of wasps, it is essential to consciously retain colonies with high production performance and strong disease resistance as the breeding population. By selectively breeding queens and males, and through multiple generations of selective breeding, the disease resistance of wasps can be gradually enhanced. Alternatively, new disease-resistant varieties can be developed through hybrid breeding, which can elevate the disease resistance level of wasps. In recent years, the widespread application of artificial insemination technology in bee breeding has also provided a technical means for the breeding of disease-resistant wasp strains [35]. Through artificial insemination, the genetic combination of wasps can be more precisely controlled, thereby increasing the probability of inheritance of disease-resistant traits. An ideal disease-resistant variety should possess characteristics such as high yield, good quality, and high egg-laying capacity of the queen.

#### 4.3.3. Management Techniques for Prevention

Scientific rearing and management techniques are essential for the healthy development of wasp colonies and for enhancing their disease resistance. As ectothermic animals, wasps’ body temperatures fluctuate with changes in the external environment. Prolonged exposure to temperatures between 40 and 50 °C can disrupt their metabolic balance, shorten their lifespan, and ultimately lead to death. In high-temperature environments (above 30 °C), measures such as increasing ventilation, providing shade, or spraying water around the wasp nests are necessary to reduce temperatures. Therefore, stable temperature management is essential for their healthy development and disease resistance. The optimal activity temperature range for wasps is 23–30 °C. An ample supply of wasp food is crucial for maintaining the health and efficient reproduction of wasps. Starved wasp colonies are often the initial groups to suffer from diseases. Wasp food not only provides the necessary energy source for wasps but also supplements key nutrients such as vitamins and salts. Therefore, paying attention to comprehensive nutritional feeding can continuously improve the physical condition of wasp colonies and enhance their disease resistance. A high-quality queen is also one of the important reasons for achieving high yields. New queens typically have strong vitality and vigorous metabolism, which often results in a lower pathogen load compared to older queens. This reduced pathogen carriage endows new queens with enhanced disease resistance. For instance, in the control of sacbrood disease, queen replacement is an important integrated control measure. By replacing the queen, it is possible to ensure that 1 to 2 generations of offspring do not contract the disease or have only a few larvae affected. The living conditions of wasps are also crucial for their survival and reproduction. Wasps generally prefer relatively enclosed, well-insulated, and dark environments. The quality and integrity of the wasp nests box significantly impact the maintenance of a suitable internal environment. If the nests have gaps or are incomplete, leading to air leakage, it becomes difficult to maintain a stable internal temperature. This instability can prompt wasps to engage in excessive activity to generate heat, thereby increasing energy consumption. Prolonged exposure to such conditions can weaken the wasps’ resistance, making them more susceptible to external environmental pressures and pest infestations.

## 5. Product Harvesting, Processing, and Storage

During the final reproductive period of wasps from July to September, the pupae attain their maximum size and peak nutritional value, making this the optimal time for harvesting. Harvesters should wear professional anti-sting suits and choose to harvest at night when workers are inactive. It is also important to retain the queen and some workers to ensure the continuation of the colony [23]. Adult and larval wasps are collected, blanched in boiling water, de-winged, and then dried either by sun-drying or using a drying oven for storage [3]. During harvesting, the integrity of the nest should be maintained as much as possible. After removing dead insects and impurities, the nest is subjected to sun-drying. The pupae should be rinsed and blanched in saltwater, then dried. Subsequently, they should be vacuum-packed and stored at low temperatures. After the comb is dried, it should be placed in a well-ventilated and moisture-proof environment, with regular inspections conducted to prevent infestation.

## 6. Composition, Extraction, and Application of Wasp Venom

The venom of wasps possesses significant medicinal value. It is a colorless liquid that turns into a light-yellow powder when dried, containing 26% solid matter, of which 76% is protein. It is mainly composed of amines, peptides, enzymes, and other proteins [42]. The venom of wasps is stored separately in the acid and alkaline glands of the sting apparatus of female wasps, and it comes in two types: acidic and alkaline. In traditional medicine, wasp venom has been used to treat rheumatoid arthritis and cardiovascular diseases [6]. Modern research has shown that the components of wasp venom have antibacterial, anticancer, anti-inflammatory, and anticoagulant effects [43,44,45]. For instance, the protein components of the venom of *V. mandarinia* have significant anti-inflammatory and anti-aging effects [46]. The venom of wasps has the ability to manage synovial inflammation and provide treatment for RA; moreover, it downregulates the expression of inflammatory factors induced by tumor necrosis factor-α and enhances apoptosis [47,48]. The peptide components of wasp venom, such as melittin, exhibit strong hemolytic properties and have demonstrated anti-rheumatic, anti-rheumatoid arthritis, antibacterial, antiviral, hypotensive, and anticancer activities [6,49,50,51,52]. In the field of nanotechnology, the development of bee venom nanoemulsions has been shown to exhibit anti-inflammatory effects [53]. However, the low yield and high cost of venom, coupled with the immaturity of wasp rearing techniques and venom extraction methods, make it difficult to control the quality of raw materials. Traditional methods involve manually extracting venom from live wasps, which is both time-consuming and risky. Modern methods include using electrical stimulation to induce venom release, which can increase yield but require careful operation to avoid harming the wasps [18]. After collection, impurities are removed, and the venom undergoes freeze-drying for preservation. The dried venom product should be stored in a frozen state and avoid repeated freezing and thawing. Currently, although some progress has been made in wasp rearing techniques and the functional active components of venom, it is necessary to accelerate the promotion of artificial wasp rearing techniques and the exploration of venom extraction methods. Modern biotechnology should be utilized to strengthen the screening of effective active components in wasp venom, pharmacological research, and the industrialization of the functional active components of venom that have been discovered.

## 7. Discussion

Wasps are an important group of resource insects in China, with a long history of utilization. They possess multiple values, including medicinal use, nutrition, biological control of pests, and pollination. In recent years, as the market demand for wasps and their products has increased, the technology for artificial rearing of wasps has gradually developed. Large-scale rearing bases have been established in regions such as Guizhou, Yunnan, Hunan, Sichuan, Guangdong [54]. It has been reported that in Yunnan Province, China, there are over 10,000 households engaged in wasp rearing, with a breeding quantity reaching 200,000 nests. This industry brings an average annual income increase of over 20,000 CNY per farmer, and the total output value is close to 200 million CNY [55]. Current rearing technologies have made significant progress in overcoming critical challenges such as artificial queen overwintering and pest control. These advancements significantly improve the oviposition rate of wasps, the survival rate of larvae, and the overall productivity of the colony [7]. However, these successes often remain context-specific and have not yet achieved the reliability or scalability commonplace in the well-established apiculture industry. The persistent industry bottlenecks, including a scarcity of high-quality prospective queens, the singularity of rearing species, the lack of a standardized indoor rearing system, insufficient capacity for large-scale disease control, and limitations in year-round cyclic rearing techniques. Currently, semi-domesticated and small-scale rearing remains the predominant method [55]. This stands in stark contrast to the fully domesticated status of honeybees [35]. The continued dominance of small-scale, semi-wild practices highlights a fundamental reliance on wild populations and their natural cycles, rather than on controlled, population-level management. This dependency makes the industry particularly vulnerable to seasonal fluctuations and disease outbreaks, issues that FAO guidelines for sustainable insect farming explicitly recommend mitigating through closed-cycle production and genetic stock improvement [56]. Consequently, the paramount challenge is to transition from this low-control model to the development of standardized, cost-effective indoor rearing technologies. The ultimate goal is to achieve year-round indoor rearing and even factory-style production. This shift would eliminate dependence on wild resources, thereby mitigating potential threats to local ecosystems and agricultural biodiversity, while also reducing safety risks to humans and livestock [57]. In the future, it is necessary to strengthen the screening of high-quality wasp breeds. A deeper understanding of wasp nutritional ecology is essential for formulating standardized, efficient artificial diets. It is a cornerstone of any successful mass-rearing enterprise, as demonstrated in the production of black soldier flies or mealworms. Concurrently, optimizing venom and pupae processing, coupled with rigorous pharmacological characterization, is vital for unlocking their high-value medicinal potential. Efforts should also be made to promote the application development of wasps in the fields of medicine, health products, and biological control. Furthermore, achieving industrial-scale wasp cultivation hinges on a foundation of fundamental scientific knowledge, such as their biological characteristics. It is essential to develop rational rearing protocols based on this knowledge, enhance practitioners’ systematic understanding of wasp biology and behavior, and continuously refine management models by integrating modern agricultural technologies [58]. As a typical social insect, the wasp exhibits complex and fascinating behaviors. In-depth exploration of fundamental biological questions regarding wasp nesting behavior, predatory behavior, and reproductive processes is essential for advancing scientific understanding. Only by continuously deepening scientific research and improving the technical system can we provide technological support for the rapid and healthy development of the wasp industry.

## Figures and Tables

**Table 1:** The differences between field rearing and artificial indoor rearing modes.

Comparison Dimension	Field Rearing	Indoor Artificial Rearing
Environmental control	Relies on natural climate; largely uncontrollable	Precise control over temperature, humidity, and photoperiod
Initial investment	Relatively low	Relatively high (requires construction/leasing of facilities and equipment)
Labor input	Relatively low, with extensive management	Relatively high, requiring intensive management
Impact of climate/predators	Significant (typhoons, drought, predators)	Minimal, due to environmental isolation
Queen oviposition period	Limited by natural seasonal cycles	Can be extended via environmental manipulation, enabling year-round production
Disease/pest risk	Higher risk due to increased interaction with the wild environment	Isolated environment allows for controlled risk
Product quality	Perceived as closer to wild; often higher market acceptance	Requires careful nutritional balance to prevent quality decline due to homogeneous environment
Suitable application	Suitable for initial colonization and population expansion	Suitable for large-scale production

## Data Availability

The original contributions presented in this study are included in this article.
